# Promoter expression of HERV-K (HML-2) provirus-derived sequences is related to LTR sequence variation and polymorphic transcription factor binding sites

**DOI:** 10.1186/s12977-018-0441-2

**Published:** 2018-08-20

**Authors:** Meagan Montesion, Zachary H. Williams, Ravi P. Subramanian, Charlotte Kuperwasser, John M. Coffin

**Affiliations:** 1 Department of Molecular Biology and Microbiology, Tufts University School of Medicine, Boston, MA USA; 2 Department of Developmental, Chemical, and Molecular Biology, Tufts University School of Medicine, Boston, MA USA; 3 Raymond and Beverly Sackler Convergence Laboratory, Tufts University School of Medicine, Boston, MA USA; 4 Present Address: Foundation Medicine, Inc., Cambridge, MA USA; 5 Present Address: Excerpta Medica, New York, NY USA

**Keywords:** Endogenous retrovirus, HERV-K, HML-2, LTR, Transcription, Tumorigenesis

## Abstract

**Background:**

Increased transcription of the human endogenous retrovirus group HERV-K (HML-2) is often seen during disease. Although the mechanism of its tissue-specific activation is unclear, research shows that LTR CpG hypomethylation alone is not sufficient to induce its promoter activity and that the transcriptional milieu of a malignant cell contributes, at least partly, to differential HML-2 expression.

**Results:**

We analyzed the relationship between LTR sequence variation and promoter expression patterns in human breast cancer cell lines, finding them to be positively correlated. In particular, two proviruses (3q12.3 and 11p15.4) displayed increased activity in almost all tumorigenic cell lines sampled. Using a transcription factor binding site prediction algorithm, we identified two unique binding sites in each 5′ LTR that appeared to be associated with inducing promoter activity during neoplasia. Genomic analysis of the homologous proviruses in several non-human primates indicated post-integration genetic drift in two transcription factor binding sites, away from the ancestral sequence and towards the active form. Based on the sequences of 2504 individuals from the 1000 Genomes Project, the active form of the 11p15.4 site was found to be polymorphic within the human population, with an allele frequency of 51%, whereas the activating mutation in the 3q12.3 provirus was fixed in humans but not present in the orthologous provirus in chimpanzees or gorillas.

**Conclusions:**

These data suggest that stage-specific transcription factors at least partly contribute to LTR promoter activity during transformation and that, in some cases, transcription factor binding site polymorphisms may be responsible for the differential HML-2 expression often seen between individuals.

**Electronic supplementary material:**

The online version of this article (10.1186/s12977-018-0441-2) contains supplementary material, which is available to authorized users.

## Background

Retroviruses are unique in that they are the only virus family known to exist in both endogenous and exogenous forms [1, 2]. Their integrated DNA sequences, known as proviruses, include at least four genes (*gag*, *pro*, *pol*, and *env*) flanked by long terminal repeats (LTRs), which contain all elements necessary to initiate and terminate viral transcription [2, 3]. Genetic transmission of these sequences occurs with germline integration, producing endogenous retroviruses (ERVs). ERVs are inherited in a Mendelian fashion and are subject to natural selection; those with deleterious effects are generally either lost from the population or inactivated by mutation, whereas those with neutral or advantageous effects remain [2, 4]. As a consequence of the accumulation of these elements over time, nearly 8% of the human genome is derived from such viral sequences [5–7].

Once classified with other “junk DNA”, ERVs are now credited with providing genomic plasticity through the use of viral proteins for host functions and alternative regulation of host gene transcription. For example, proviruses contain numerous promoters, splice sites, transcription factor binding sites, and polyadenylation signals, all of which can have significant effects on neighboring host gene expression [2, 8, 9]. Syncytins, fusogenic proteins derived from ERV *env* sequences, are essential for placenta development and mediate cell fusion to form the syncytiotrophoblast layer [10, 11]. Although ERV expression is usually silenced through epigenetic and chromatin modification, primarily via CpG methylation [8, 12–14], there are a few known instances of host cell co-option of ERV expression. Recent studies show human endogenous retrovirus (HERV) expression to be increased in human embryonic stem cells (hESCs) and human preimplantation embryos and to play a critical role during embryogenesis through the maintenance of pluripotency and hESC identity [15–19]. Increased expression of HERV proteins was found to be correlated with increased IFITM1 expression, resulting in viral immunoprotection during human embryogenesis [19, 20].

Despite these exceptions, increased HERV activity is largely associated with malignancy, especially cancer. Activation of stem cell-associated retroviruses (SCARs) in human cancer is hypothesized to be associated with increased likelihood of metastasis, immune evasion of cancer cells, and a predictive marker of poor prognosis [21, 22]. Increased cancer-related expression is attributable in part to global hypomethylation, a common consequence of tumorigenesis, and LTR hypomethylation is widely documented to result in promoter activation [11, 13, 23]. However, in vitro treatment with 5-aza-2′-deoxycytidine, a DNA methyltransferase inhibitor, shows that LTR hypomethylation alone is not always sufficient to induce promoter activity, suggesting that the proper transcriptional milieu of a cell may also be required [24–26]. Ubiquitous transcription factors, such as Sp1, Sp3, and YY1, are linked with LTR activity but do not explain the cell-specific expression that is often seen [8, 25, 27].

Expression from HERV-K (HML-2), the most recently integrated and biologically active HERV group, is upregulated in up to 85% of breast cancer samples, although the mechanism of activation is still unclear [28–31]. RNA sequence analysis of cells in an in vitro mammary carcinogenesis model shows that LTR-driven transcription of HML-2 proviruses is restricted to tumorigenic human mammary epithelial cells (HMECs), suggesting that stage-specific transcription factors appearing during malignant transformation play a role in LTR activation [32]. The goal of this study was to investigate how LTR sequence variation among the various HML-2 proviruses affects activation of its promoter during HMEC transformation.

Overall, we found the most widespread increase in promoter activity during transformation in two proviruses (located at 3q12.3 and 11p15.4). Through a combination of reporter construct assays and RNA-Seq analyses, we identified two transcription factor binding sites on each 5′ LTR that were associated with promoter activity in transformed cells. Further genomic analysis of these proviruses, using data from the 1000 Genomes Project as well as comparison with homologous proviruses in several other hominoid species, showed that both of these sites had been created by mutations in the 5′ LTR that occurred post viral integration. The 3q12.3 site has become fixed in the human population whereas that at 11p15.4 is polymorphic, with the active form having an allele frequency of 51%. In both cases, these sites have evolved away from the inactive ancestral sequence and towards an active form. These results emphasize the importance of studying HERV transcription at the single provirus and single nucleotide level, as polymorphisms in critical binding sites may be responsible for the differential HML-2 expression often seen between individuals.

## Results

### Differential HML-2 promoter expression is correlated with 5′ LTR sequence similarity

The HML-2 5′ LTR contains all elements necessary for driving transcription. Removal of core promoter elements results in reduced promoter activity, suggesting that these sequences are critical for proper LTR-driven expression [9, 25, 33]. Each provirus has accumulated numerous unique mutations over time, suggesting that LTR sequence variation could contribute to differential HML-2 expression, particularly through the alteration of transcription factor binding sites. Through a series of dual-luciferase assays, we sought to evaluate whether LTR sequence identity is correlated with similar promoter expression patterns during breast cancer tumorigenesis.

The proviruses of interest for this study were chosen based on a preliminary investigation in which we used single-genome sequencing to detect provirus-specific transcripts from eight human breast cancer cell lines. From this analysis, we produced a list of the top ten highest expressing HML-2 proviruses within these cell lines tested (Additional file 1). 16p11.2 and K105 were excluded from our study since 16p11.2 has no 5′ LTR and the unmapped K105 exhibited cloning inconsistencies caused by its location within the unassembled centromeric region Un_g1000219 [4, 34]. The remaining eight proviruses, plus 8p23.1c, a segmental duplication of 11p15.4 [4], were chosen as our loci of interest. The alternative names and chromosomal locations of these proviruses are listed in Table 1.

**Table 1:** HML-2 proviruses with alternative names and genomic coordinates

Provirus	Alternative names	Chromosomal location (hg19)
1q22	K102, K(C1b), K50a, ERVK-7	chr1:155,596,457–155,605,636
3q12.3	KII, ERVK-5	chr3:101,410,737–101,419,859
3q21.2	KI, ERVK-4	chr3:125,609,302–125,618,439
5p13.3	K104, K50d	chr5:30,486,760–30,496,205
7p22.1b	K108R, ERVK-6	chr7:4,630,561–4,640,031
8p23.1c		chr8:12,073,970–12,083,497
11p15.4	K7	chr11:3,468,656–3,478,209
21q21.1	K60, ERVK-23	chr21:19,933,659–19,941,962
22q11.21	K101, K(C22), ERVK-24	chr22:18,926,187–18,935,361

From Subramanian et al. [4] and Montesion et al. [32]

**Fig. 1:**
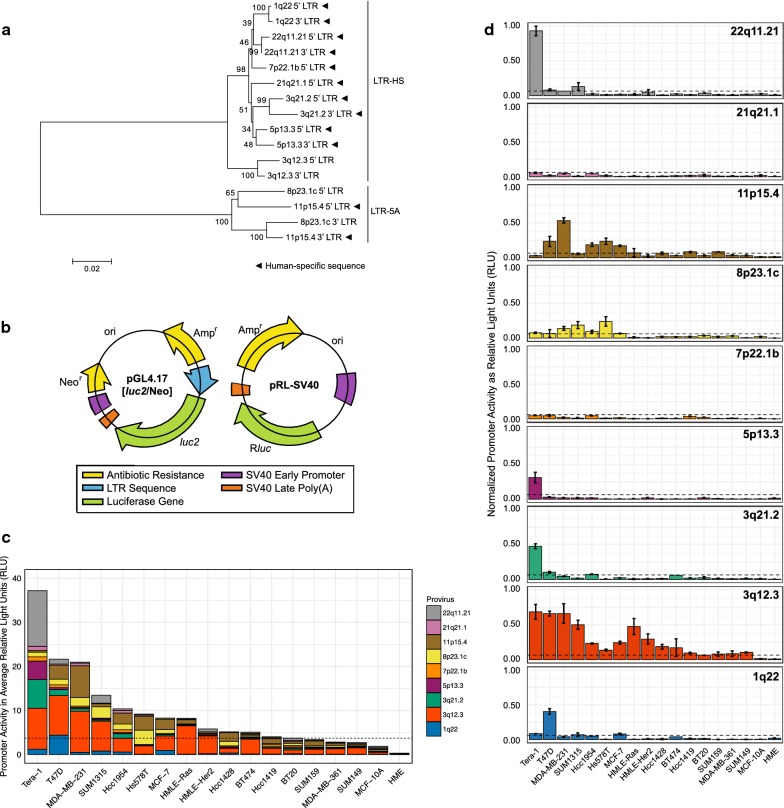
HML-2 proviruses exhibit differential promoter activity in tumorigenic cells and negligible activity in immortalized HMEs. **a** Neighbor-joining tree displaying 5′ and 3′ LTR sequence relationship of the nine HML-2 proviruses used in this study. Bootstrap values are shown to the left of each node and scale is substitutions/site. LTR type (LTR-HS or LTR-5A) is shown to the right of the tree. Human-specific sequences are designated with a black triangle. **b** Schematic of the reporter constructs used in the dual-luciferase assay. Left, promoter-less firefly luciferase vector (pGL4.17[*luc2*/Neo]). Right, control *Renilla* luciferase vector (pRL-SV40). Direction of gene transcription is shown by arrows. Important gene regions are differentiated by colors and the names associated with those colors are displayed underneath. **c**, **d** Relative 5′ LTR promoter activity determined by dual luciferase assay in eighteen human cell lines. Data are organized by cell line in **(c)** and by provirus in (**d**). Promoter activity is displayed as relative light units (RLU) normalized against the internal control *Renilla* expression. Data in (**d**) are normalized against the highest expression value in the dataset. Statistical significance (dashed line, p < 0.05) was generated by ANOVA with Bonferroni’s multiple comparisons test and is based on comparisons to HME expression. All experiments were conducted in triplicate and data displayed as mean (**c**) or mean ± standard deviation (**d**)

Phylogenetic analysis of the LTRs from these nine proviruses shows that most of them are classified as LTR-HS, the LTR group that contains the youngest proviruses, including ~ 90% of the human-specific integrations (Fig. 1a) [4, 9, 35]. The 5′ LTR sequences from each provirus were cloned into pGL4.17[*luc2*/Neo], a promoter-less firefly luciferase vector, directly upstream of the *luc2* gene. The relative promoter activity of these sequences was determined based on *luc2* expression and normalized against that of an internal control vector, containing a *Renilla* luciferase gene (R*luc*) driven by an SV40 promoter (Fig. 1b). A panel of eighteen human cell lines was transiently co-transfected with these vectors. The panel comprised of two immortalized HMEC cell lines, fifteen tumorigenic breast cancer cell lines (representing all three molecular subtypes), and one teratocarcinoma cell line known to produce HML-2 transcripts and retroviral-like particles (RVLPs) at high levels [9, 36]. Characterization of the cell lines used is shown in Table 2.

**Table 2:** Characterization of cell lines used for transfection

Breast cancer molecular subtype	Hormone receptor status	Cell lines
Luminal	ER+ and/or PR + HER2 ±	T47D, MCF-7, Hcc1428, BT474, MDA-MB-361
HER2/*neu*	ER− PR− HER2 +	SUM1315, Hcc1954, Hcc1419
Basal	ER− PR− HER2 −	MDA-MB-231, Hs578T, BT20, SUM159, SUM149

Additional cell types	Cell lines
Immortalized human mammary epithelial cells	HME, MCF-10A
Transformed human mammary epithelial cells	HMLE-Her2, HMLE-Ras
Human teratocarcinoma cells	Tera-1

Although minimal promoter activity was detected in immortalized HMECs transfected with any of the HML-2 LTR reporter constructs, significant upregulation of expression driven by one or more LTRs was seen in 73% (11/15) of the tumorigenic breast cancer cell lines (Fig. 1c). This expression pattern is consistent with previous reports that suggest up to 85% of breast cancer samples have a significant increase in HML-2 activity [29, 31, 37]. Overall, each LTR was significantly expressed in at least one cell line tested but showed differential expression across the panel. Two proviruses (3q12.3 and 11p15.4) were significantly upregulated in nearly all neoplastic cell lines investigated, whereas others were only upregulated in a select few (Fig. 1d). The highest level of combined HML-2 expression in a breast cell line was exhibited by T47D (Fig. 1c), a tumorigenic breast cancer cell line known to produce RVLPs under hormonally-stimulated conditions [3, 38, 39]. However, this activity level was only about half that seen in the Tera-1 cells, consistent with our previous report that Tera-1 cells produce markedly higher numbers of HML-2 transcripts than breast cancer cell lines [32].

**Fig. 2:**
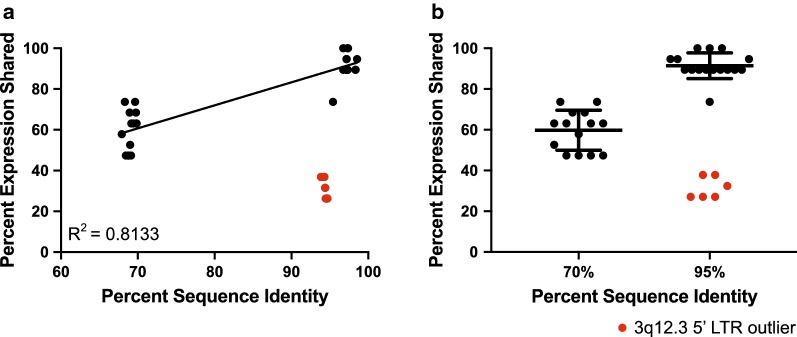
LTR sequence identity is correlated with promoter expression patterns, with the exception of 3q12.3. Scatter plots displaying the correlation between percent sequence identity and shared percent expression. Raw values are shown in Additional file 2 and are based on pairwise comparisons. Best fit line and its R^2^ value are shown for (**a**) (black values only). Error bars depict the mean ± standard deviation in (**b**) (black values only). Outlying 3q12.3 5′ LTR data points are shown in red for both plots

We next sought to determine if LTRs of similar sequence share similar patterns of promoter activity. For this purpose, we created a percent sequence identity matrix, by multiple sequence alignment using Clustal Omega [40], and an HML-2 percent expression similarity matrix, determined through pairwise comparisons of significant promoter activity within each cell line tested (Additional file 2). We found the two values to be correlated, suggesting that LTRs with high sequence similarity are more likely to exhibit significant promoter activity under the transcriptional environment of the same cell line (Fig. 2a). Overall, LTRs with ~ 70% sequence similarity shared promoter expression patterns ~ 60% of the time, whereas LTRs with ~ 95% sequence identity shared promoter expression patterns ~ 90% of the time (Fig. 2b). With the exception of the 5′ LTR of 3q12.3 (Fig. 2, red), the sequences clustered into two observable groups. The expression pattern of the 3q12.3 5′ LTR was not similar to any other LTR and instead exhibited unusually high promoter activity levels, with significant promoter expression seen in almost every transformed cell line investigated (Fig. 1d).

### Identification of transcription factor binding sites critical for HML-2 promoter activity during neoplasia

The association between LTR sequence and cell line-specific expression suggests that certain sequence-specific elements, such as transcription factor binding sites, play a large role in determining differential promoter activity. Increased HML-2 expression is largely seen during tumorigenesis and our recent results indicate that LTR-driven transcription does not occur until post-transformation [32]. The following experiments were performed to further investigate the relationship between malignant transformation and expression and to elucidate the specific LTR sequences required.

For this purpose, we focused on three cell lines: HME, HMLE-Her2, and HMLE-Ras. These cells were all derived from the same HMEC population and are therefore isogenic, differing only by oncogene overexpression. HME cells are non-transformed but immortalized through hTERT (human telomerase reverse transcriptase) overexpression. The HMLE cells, in addition to being hTERT-immortalized, are transformed through the introduction of SV40 large and small T antigens. HMLE-Her2 and HMLE-Ras differ from one another by their oncogene overexpression, *ERBB2* (also known as HER2/*neu*) and *HRAS*, respectively. These cell lines provided the opportunity to investigate how specific differences in the transcriptional environment of the cell can affect LTR expression.

**Fig. 3:**
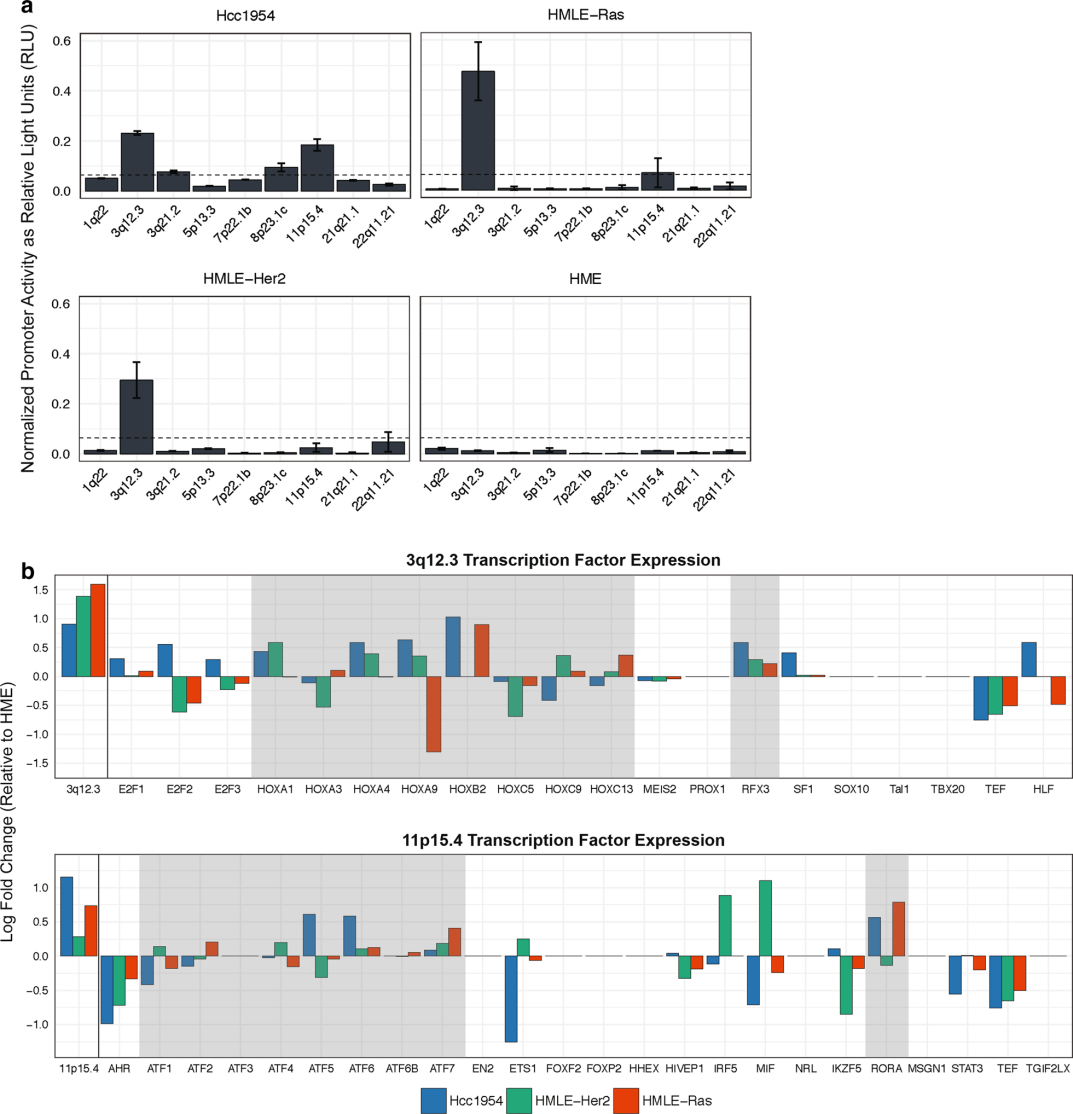
Identification of transcription factor binding sites critical for tumorigenic HML-2 promoter activity. **a** Relative 5′ LTR promoter activity in the tumorigenic Hcc1954, HMLE-Ras, and HMLE-Her2 cell lines as well as the immortalized HME cell line. Promoter activity is determined as relative light units (RLU) normalized against the internal control *Renilla* luciferase expression and normalized against the highest expression value in the dataset. Statistical significance (dashed line, p < 0.05) was assessed by ANOVA with Bonferroni’s multiple comparisons test and is based on comparisons to HME expression. All experiments were conducted in triplicate and data displayed as the mean ± standard deviation. **b** The log fold change of relative transcript abundance levels, in FPKM, of transcription factors predicted to bind to unique sites on the 5′ LTR of 3q12.3 (top) and 11p15.4 (bottom). Fold change is relative to expression in the HME cell line. The log fold change of relative promoter activity (RLU) in the 5′ LTR of 3q12.3 and 11p15.4 is shown to the left of each respective plot. Highlighted in gray are the transcription factors known to bind to the HOX-PBX and RFX3 binding sites (top) as well as the transcription factors known to bind to the ATF and RORA binding sites (bottom)

We detected increased promoter activity from 3q12.3 and 11p15.4 in HMLE-Ras cells as well as increased activity from 3q12.3 in HMLE-Her2 cells. The significance of this expression was determined as compared to the HME cell line (Fig. 3a). In effort to explain this pattern, we sought to identify transcription factor binding sites that are unique to each LTR and therefore may be responsible for the selective activation seen of one LTR over another. Using MatInspector, a transcription factor binding site prediction software by Genomatix [41], we found a total of 63 unique sites among the nine LTRs in this study. Of those, 13 were unique to 3q12.3 and 20 were unique to 11p15.4 (Table 3).

**Table 3:** Unique transcription factor binding sites found in HML-2 5′ LTRs of interest

Provirus	Unique binding site
1q22	NBRE^‡^
3q12.3	CDE, E2F, HOX-PBX, MRG1^‡^, PROX1, RFX3^†^, SF1^†^, SOX10, TAL1-E2A, TBX20, TEF-HLF^‡^, TGIF^†^, TR2^‡^
3q21.2	GLI1^†^, IK3, NFY^‡^, NKX29^†^, SIX2^‡^, STAT5
5p13.3	CARF^‡^, MYBL1^‡^
7p22.1b	EKLF^‡^, GAGA^‡^, GLI3^‡^
8p23.1c	AML1^‡^, BHLHB2^‡^, DMRT7^‡^, HMGA^‡^, HOX1-3^‡^, MAFF^‡^, MEF2^‡^, NRF1^‡^, PAX1^‡^, SOX17^‡^, STAT5A^‡^
11p15.4	AHRARNT^‡^, ATF, ATF6, CETS1P54, EN2^‡^, ETS1, FOXP2^†^, FREAC2^‡^, HDBP1-2, HHEX, HIVEP1^†^, IRF5, MIF1^†^, NRL, PEGASUS, RORA, SGN1, STAT3, TEF^†^, TGIF2LX
21q21.1	CHOP^†^, NFKAPPAB50^†^, USF^†^, ZNF300^†^
22q11.21	GRHL1^‡^, MASH1^†^, TAL1BETAHEB^†^

Only sites unique to each 5′ LTR, as compared to the other eight 5′ LTRs, are shown

^†^Present only in other HML-2 solo LTR(s)

^‡^Present in other HML-2 full length provirus(es) and solo LTR(s)

The same software was used to create a list of transcription factors predicted to bind to the unique sites on the 5′ LTRs of 3q12.3 and 11p15.4. In a previous study [32] the expressed RNAs of the HMLE-Ras, HMLE-Her2, and HME cell lines were sequenced, alongside the established human breast cancer cell line Hcc1954, using Illumina MiSeq sequencing. The transcript abundance levels, measured as FPKM, of these transcription factors were compared to assess upregulation of their expression in the tumorigenic cell lines as compared to the non-transformed HME control, and related to levels of expression of the proviruses at 3q12.3 and 11p15.4. Overall, we saw a significant increase in expression of transcription factors known to bind to the HOX-PBX and RFX3 sites on the 3q12.3 5′ LTR as well as a significant increase in those known to bind to the ATF and RORA sites on the 11p15.4 5′ LTR (Fig. 3b), implicating these sites and one or more of the upregulated factors in LTR activation during neoplasia.

### Removal of critical binding sites decreases HML-2 promoter activity in neoplastic cell lines

**Fig. 4:**
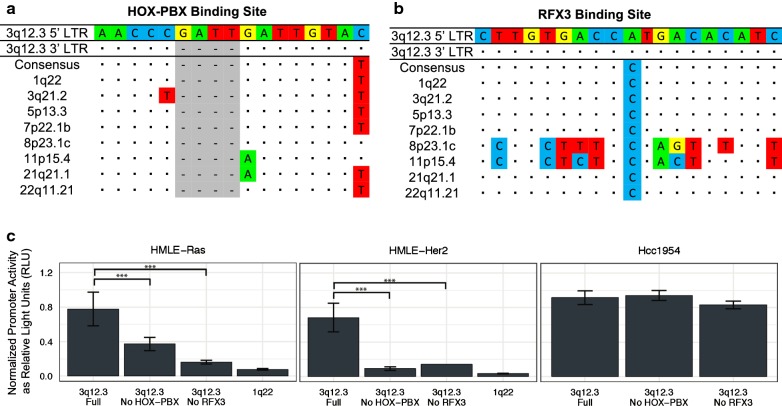
Back mutation of critical transcription factor binding sites to consensus sequences on the 3q12.3 provirus. **a**, **b** Multiple sequence alignment of the **a** HOX-PBX and **b** RFX3 binding regions on the nine 5′ LTRs of interest in this study as well as a consensus sequence of the site. Sequences are compared against the 3q12.3 5′ LTR site, dots are used for shared identity, and dashes and shading indicate indels. **c** Relative 5′ LTR promoter activity in HMLE-Ras cells, HMLE-Her2 cells, and Hcc1954 cells. Constructs used either contained full HOX-PBX and RFX3 binding sites, or had a binding site removed through back mutation to the consensus sequence. Promoter activity of the 1q22 5′ LTR is shown for comparison. Promoter activity is determined as relative light units (RLU) normalized against the internal control *Renilla* luciferase expression. Statistical significance was assessed by ANOVA with Bonferroni’s multiple comparisons test (***p < 0.0005). All experiments were conducted in triplicate and data are display as the mean ± standard deviation

The functionality of these sites was assessed by mutating each one individually. A multiple sequence alignment was performed using the sequences of all nine 5′ LTRs. From this analysis, we created a consensus sequence for each critical binding site, which we deemed to be the “non-active” version of each site. The full binding site sequence in each 5′ and 3′ LTR of the nine proviruses of interest in this study are provided in Additional file 3, Additional file 4, Additional file 5 and Additional file 6. The 3q12.3 HOX-PBX binding site differed from the consensus non-active sequence by a five base pairs, including a duplication of four nucleotides (Fig. 4a). Reversion of these sites significantly decreased LTR promoter activity in both neoplastic cell lines, with activity decreasing by twofold in HMLE-Ras cells (Fig. 4c, left) and by sevenfold in HMLE-Her2 cells (Fig. 4c, middle). The 3q12.3 RFX3 binding site only differed from the consensus sequence by one nucleotide, an A to C transversion (Fig. 4b), and yet removal of this site decreased LTR activity by fivefold in both HMLE-Ras cells (Fig. 4c, left) and HMLE-Her2 cells (Fig. 4c, middle). Activity was decreased to levels comparable to that of 1q22, a proviral LTR with no significant promoter activity in these cell lines (Fig. 4c). Mutating these sites did not significantly decrease LTR promoter activity in Hcc1954 cells (Fig. 4c, right), which also showed elevated expression of transcription factors known to bind to five unique 3q12.3 sites (E2F, HOX-PBX, RFX3, SF1, TEF-HLF) (Fig. 3b), suggesting that the other active binding sites can compensate for promoter activity when only some of them are removed.

**Fig. 5:**
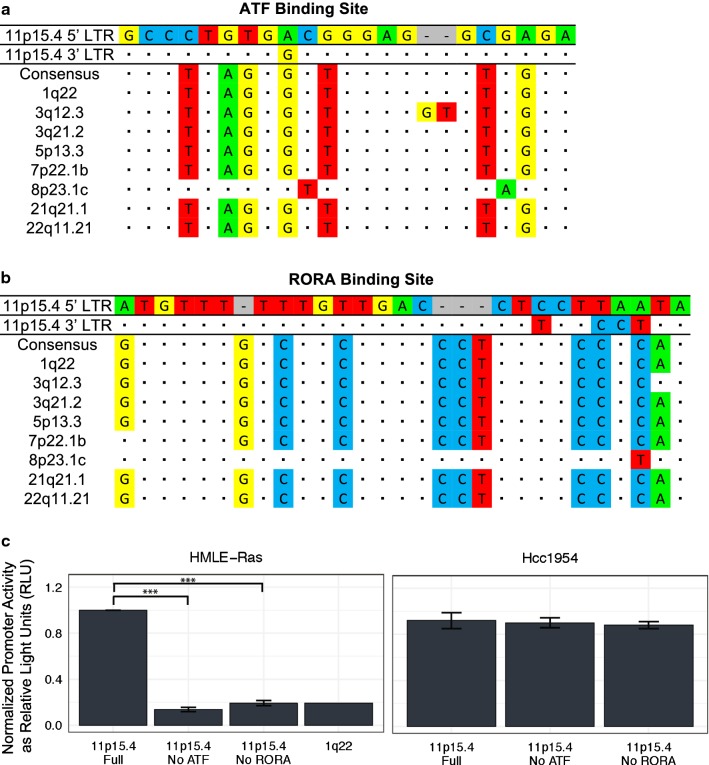
Back mutation of critical transcription factor binding sites to consensus sequences on the 11p15.4 provirus. **a**, **b** Multiple sequence alignment of the **a** ATF and **b** RORA binding regions on the nine 5′ LTRs of interest in this study as well as a consensus sequence of the site. Sequences are compared against the 11p15.4 5′ LTR site, dots are used for shared identity, and dashes indicate indels. **c** Relative 5′ LTR promoter activity in HMLE-Ras cells and Hcc1954 cells. Constructs used either contained full ATF and RORA binding sites, or had a binding site removed through back mutation to the consensus sequence. Promoter activity of the 1q22 5′ LTR is shown for comparison. Promoter activity is determined as relative light units (RLU) normalized against the internal control *Renilla* expression. Statistical significance was generated by ANOVA with Bonferroni’s multiple comparisons test (***p < 0.0005). All experiments were conducted in triplicate and data displayed as the mean ± standard deviation

Similar results were seen with the 11p15.4 5′ LTR. The consensus sequence differed from the ATF binding site by nine nucleotides (Fig. 5a) and back mutating the binding site to match the consensus sequenced decreased promoter activity by sixfold in HMLE-Ras cells (Fig. 5c, left). The RORA binding site differed by eleven nucleotides from the consensus sequence (Fig. 5b) and mutating all of these to the consensus bases decreased promoter activity by fivefold in HMLE-Ras cells (Fig. 5c, left). Again, these changes decreased activity to levels comparable with 1q22 (Fig. 5c, left). As in the case of 3q12.3, no decrease in promoter activity was seen in the Hcc1954 cell line (Fig. 5c, right), which had elevated expression of transcription factors known to bind to four unique 11p15.4 sites (ATF, HIVEP1, PEGASUS, RORA) (Fig. 3b, bottom).

### Most unique HML-2 transcription factor binding sites were acquired over time following integration and are fixed in the human population

**Fig. 6:**
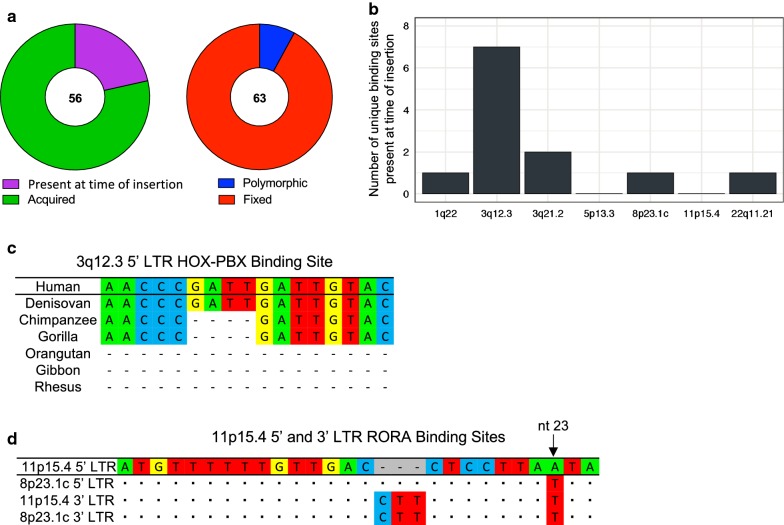
Characterization of unique transcription factor binding sites. **a** Pie charts describing the unique binding sites in the 5′ and 3′ LTRs of proviruses in this study. Shown is the number of unique binding sites that were either present at the time of insertion or acquired over time (left) as well as the number of unique binding sites that are either polymorphic or fixed in the human population (right). **b** The number of unique transcription factor binding sites identified as being present at the time of insertion found on each 5′ LTR of interest in this study. **c** Multiple sequence alignment of the human HOX-PBX binding site in the 3q12.3 5′ LTR as compared to the homologous sequences in different non-human primate reference genomes. Dashes indicate indels. **d** Multiple sequence alignment of the RORA binding site in the 11p15.4 5′ and 3′ LTRs as compared to the 5′ and 3′ LTRs of the 8p23.1c provirus. Dots are used for shared identity and dashes indicate indels. The nucleotide that appears to be responsible for the RORA functional polymorphism, is shown with an arrow

At the time of integration, the 5′ and 3′ LTRs of a provirus are almost always identical. Over time, as mutations are accumulated, sequence variation between the two LTRs increases. By aligning the 5′ and 3′ LTRs of 3q12.3 and 11p15.4, we were able to determine whether these critical transcription factor binding sites were present at the time of insertion (as evidenced by its presence in both LTRs) or were acquired over time (and found in only one LTR). We determined that one of the sites, RFX3 found in 3q12.3, was present at the time of insertion, but that three of the binding sites were acquired over time (Table 4). We analyzed the remaining unique binding sites in this same manner, with the exception of sites found on 7p22.1b and 21q21.1, which do not have full 3′ LTRs. Overall, only 21% (12/56) of the unique sites were present at the time of insertion (Fig. 6a, left), the majority of which (58%, 7/12) were found in the 3q12.3 5′ LTR (Fig. 6b).

**Table 4:** Characterization of LTR binding sites critical for 3q12.3 and 11p15.4 promoter activity in tumorigenic cells

Provirus	Binding site	LTR	Binding site allele frequency	Binding site evolution
3q12.3	HOX-PBX	5′ LTR	99.68% (fixed)	Acquired
	RFX3	5′ and 3′ LTR	99.96% (fixed)	Present at the time of insertion
11p15.4	ATF	5′ LTR	99.88% (fixed)	Acquired
	RORA	5′ LTR	50.76% (polymorphic)	Acquired

To determine the distribution of these sites within the human population, we analyzed the VCF (Variant Call Format) files of 2504 individuals, as supplied by phase 3 of the 1000 Genomes Project [42]. Of the four binding sites that we found to be critical for HML-2 promoter expression during neoplasia, three had allele frequencies > 99% and are therefore fixed in the population. The RORA binding site, found in the 11p15.4 5′ LTR, was found to be polymorphic with an allele frequency of 50.76% (Table 4). Overall, only 8% (5/63) of the unique sites that we identified were polymorphic in the human population (Fig. 6a, right).

### Evolution of the HML-2 HOX-PBX and RORA binding sites

Alignment of the 5′ and 3′ LTRs of the 3q12.3 provirus revealed a 4 bp insertion, found in the middle of the HOX-PBX site, resulting from duplication of a GATT sequence (Fig. 4a). This provirus is estimated to have integrated ~ 10 million years ago and is present in gorillas, chimpanzees, and bonobos, as well as humans [4]. Using the UCSC Genome Browser, we examined this LTR in several non-human primate reference genomes. We found that despite the conservation of the 3q12.3 provirus across multiple hominoid species, the 4 bp insertion, and consequently the HOX-PBX binding site, is only present in humans and Denisovans (Fig. 6c). These results suggest that this binding site was acquired sometime after the human-chimpanzee evolutionary split and has been stably integrated in the human genome ever since.

The RORA binding site on 11p15.4 was one of the only polymorphic unique binding sites that we identified. This polymorphism is due to a single nucleotide change, where 51% of alleles in the human population contains an A at the 23rd base pair in the site (and therefore an intact RORA site) and 49% of the population contains a T. This provirus is of particular interest because 11p15.4 is a segmental duplication of 8p23.1c, which is estimated to have integrated ~ 20 million years ago. Although the proviral sequence is quite old, the duplication occurred after the human-chimpanzee split, and the 11p15.4 sequence is human-specific [4]. We aligned the 5′ and 3′ LTRs of these two proviruses and compared their sequences at the RORA binding site. We found that although both of the 3′ LTRs at this site are identical, the 5′ LTRs differ by one nucleotide, the same 23rd nucleotide that is responsible for the RORA polymorphism (Fig. 6d). The 5′ LTR also differed from the 3′ by deletion of 3 bp, which must have predated the segmental duplication of this provirus, as it was also found in the 8p23.1c 5′ LTR. Based on these observations, it appears as though the provirus at 11p15.4 in half of the human population has evolved away from the ancestral 8p23.1c sequence, resulting in a functional RORA binding site.

## Discussion

Post-integration, retroviral sequences are transcribed and translated like any other cellular gene and are subject to the same selective pressures. Germline sequences with neutral or advantageous effects can become fixed in the population, resulting in endogenization [2, 25, 43]. These sequences provide unique opportunities to study the evolutionary relationship between host and pathogen, including adaptations for assimilation within the host genome.

The full biological significance of HERVs remains to be uncovered. Repetitive mobile sequences are often credited with contributing to genome plasticity and HERVs, equipped with multiple splice junctions, promoter/enhancer sites, and polyadenylation signals, are abundantly capable of altering host gene expression [2, 8, 43]. A number of endogenous retroviruses, including the mouse mammary tumor virus, murine leukemia virus, and Jaagsiekte sheep retrovirus, exhibit both endogenous and exogenous transmission and are capable of inducing carcinogenesis. Since the pathogenicity of these viruses is generally due to LTR activity and integration site, which can result in the alteration of expression of nearby proto-oncogenes [43, 44], endogenous viral sequences are often silenced through epigenetic and chromatin modifications such as CpG methylation [12–14].

Our group recently characterized the HML-2 transcriptome during HMEC transformation and found that the site of proviral integration is often crucial for expression, with the majority of expressed proviruses being transcribed by non-LTR-driven mechanisms such as read-through from adjacent promoters. When it was present, LTR-driven transcription was detected only in tumorigenic cells, suggesting that the altered transcriptional milieu of a transformed cell is critical for LTR promoter activation [32]. The goal of this study was to investigate the interplay between LTR sequence variation and cellular environment and to look for evidence of evolutionary adaptations that could result in increased activity during neoplasia.

LTR hypomethylation, commonly seen in malignant cells, is well documented to result in increased ERV expression [13, 29, 45]. To eliminate this issue, we decided to investigate the relationship between LTR sequence similarity and differential expression patterns using reporter construct assays, where methylation status is not a factor. We chose to study nine HML-2 proviruses, shown by single-genome sequencing to be highly transcribed across a number of breast cancer cell lines (Additional file 1). Phylogenetic analysis of these 5′ LTRs classified most of them as LTR-HS, the LTR group that includes the youngest proviruses and most human-specific integrations [4, 9]. Of these, only one provirus (3q12.3) is known to not be human-specific, as it is present in gorillas and chimpanzees as well (Fig. 1a). All proviruses in this study are fixed in the human population, although one provirus (7p22.1b) is considered to be allelically polymorphic. It is present as either a solo LTR, formed through the recombination of the 5′ and 3′ LTRs and excision of the internal proviral sequence, or a full (“2-LTR”) provirus [4, 7]. However, in either case, the 5′ LTR of interest is fixed and as such, for the purpose of this study, we do not consider any of these LTRs to be insertionally polymorphic.

Overall, we found significant HML-2 promoter activity in 73% (11/15) of tumorigenic HME cell lines (Fig. 1c), consistent with previous reports of increased HML-2 expression in up to 85% of breast cancer samples [30, 31, 37]. Molecular subtype, as denoted by hormone receptor status, of the cell lines was noted (Table 2), but no significant correlation with HML-2 promoter expression was observed (Additional file 7). Pairwise comparisons of 5′ LTR sequence identity and promoter expression in our luciferase panel revealed a positive correlation between the results of the two assays (Fig. 2). These results suggest that LTRs with similar sequences share similar promoter expression patterns, most likely due to conservation of the same transcription factor binding sites and core promoter elements.

To further investigate the importance of sequence variation on LTR promoter activity, we used MatInspector, a transcription factor binding site prediction software, to generate a list of all binding sites unique to each of the nine LTRs used in this study (Table 3). We considered unique sites to be candidates for sequence variation that may explain why one LTR would be activated under a certain cellular condition instead of another. Two proviruses, 3q12.3 and 11p15.4, exhibited the highest levels of promoter activity across our luciferase panel (Fig. 1d). We used the MatInspector data, alongside RNA-Seq results from a previously published experiment by our group [32], to identify upregulated transcription factors known to bind to the unique binding sites on these two LTRs. These results provided us with two candidate sites per 5′ LTR for the promoter activation we saw during neoplasia: the HOX-PBX and RFX3 sites on 3q12.3 and the ATF and RORA sites on 11p15.4 (Fig. 3b). Removal of these sites individually decreased LTR promoter activity in HMLE-Ras and HMLE-Her2 cells by two to sevenfold (Figs. 4c, 5c).

All four of these binding sites are known to be involved with transcriptional activation, particularly during the regulation of human embryogenesis [46–49]. Interestingly, this observation is consistent with previous literature suggesting that HERVs are regulated in manners similar to stem cell genes, relying on cell-specific transcription factors and epigenetic modifications rather than TATA boxes or other canonical promoter elements [25, 33]. The evolution of the HOX-PBX and RORA binding sites were of most interest. Although the 3q12.3 provirus can be traced back through the primate lineage to gorillas, the HOX-PBX binding site is only found in the 5′ LTR in Denisovan and human genomes (Fig. 6c). Due to lack of coverage of the Neandertal reference genome at this location, it’s unclear if this binding site is present in that species. This site, created by duplication of a GATT sequence, appears to have been acquired after the evolutionary split between humans and chimpanzees and has been fixed in the human population ever since. This analysis suggests that although the 3q12.3 provirus is evolutionarily conserved amongst several non-human primate species, the HOX-PBX binding site is human-specific. Although HOX proteins are widely expressed during development, aberrant expression has been documented during malignancy and increased HOX gene expression is being investigated as a potential breast cancer biomarker [50].

Alignments between the 5′ and 3′ LTR of proviruses shed light on the evolution of unique transcription factor binding sites. We were able to determine if sites were present at the time of insertion (present in both LTRs) or acquired over time (present in only one LTR). Only 21% of the unique binding sites that we identified were present at the time of insertion (Fig. 6a, left), implying that the expression patterns observed for these proviruses would not have reflected those of the ancestral virus that gave rise to them. Furthermore, the majority of unique sites were in the 3q12.3 5′ LTR (Fig. 6b). This distribution is consistent with the greater genetic distance and greater age of this provirus from the rest of the LTR-HS group (Fig. 1a). The high degree of unique sites present at the time of insertion may also explain why this particular provirus had an expression pattern widely different from the other LTRs in this study (Fig. 2).

Due to their possible role in pathogenicity, it is essential to study the genetic differences of HML-2 elements among individuals. Most often, such studies focus on whole proviruses, studying insertional polymorphism and its possible contribution to disease. Thus far, however, no polymorphic proviruses have been found to play a role in the genesis of cancer [34, 51]. To our knowledge, ours is the first study to investigate genetic differences at the single nucleotide level, by examining SNPs within LTRs. Of the 63 binding sites unique to one of the expressed LTRs that we identified, only five of them were found to be polymorphic within the 2504 genomes mined (Fig. 6a, right). These allele frequencies were further broken down by super-population, showing only slightly higher prevalence of these binding sites in the African population (Additional file 8).

The RORA binding site, harbored on the 5′ LTR of the 11p15.4 provirus, was the only site critical for HML-2 activation during neoplasia that was also polymorphic (Table 4). This provirus is of particular interest because it is a segmental duplication of 8p23.1c [4], which showed no LTR activity during tumorigenesis. After examining the RORA binding sites on both of these LTRs, we found that 51% of the population contains an active RORA site whereas the other half of the population contains an inactive RORA site, identical to the ancestral 8p23.1c 5′ LTR. Thus, more than half of the human population has evolved away from the ancestral sequence and towards a more active LTR version (Fig. 6d).

## Conclusions

The role, if any, of HERV activity during tumorigenesis is unknown. It is currently unclear if HML-2 expression is an ancillary consequence of transformation or if it somehow aids in the event; although recent work shows that Env protein expression may increase the ability of tumor cells to evade immune surveillance during some cancers [52] or even participate directly in the transformation process by interacting with cellular proto-oncogenes [53]. Although no provirus of interest in our study is believed to have a viable open reading frame for any viral gene, protein production in these cell lines as well as any sample used in future investigations, should be examined. Our results show that HML-2 promoter activity is present in the majority (73%) of breast cancer cell lines tested and that LTR sequence similarity is correlated with promoter expression patterns. From there, we were able to map binding sites seemingly crucial for HML-2 promoter expression during neoplasia, many of which were acquired over evolutionary time. The polymorphism of certain sites provides another dimension in regards to what causes differential expression of ERVs between individuals. These data may shed light on adaptive co-evolution of ERVs within their host cells.

In recent years, there have been numerous reports of co-option of endogenous proviral sequences to disparate features of normal human and vertebrate biology, including protection against infection by related exogenous viruses [54], formation of the placental syncytiotrophoblast layer [10], expression of salivary amylase [55], stimulation of innate immunity [56], stimulation of neurological synapses promoting long-term memory [57], among others. It is particularly noteworthy that transcription of the two most highly expressed proviruses in our panel of ex vivo transformed cancer cell lines was facilitated through binding sites that were created by mutations in the 5′ LTRs that arose and spread in the human population following integration, implying that the expression patterns observed do not reflect those of the ancestral virus. It is tempting to speculate that responsiveness of the mutant proviruses to common, development-specific transcription factors might have given them some beneficial property along the lines of the ones listed above, thereby providing a selective advantage to the individuals carrying them and promoting their rapid fixation in the population.

## Methods

### Cell culture

The HME, HMLE-Her2, HMLE-Ras, MCF-10A, SUM149, SUM159, MDA-MB-361, Hcc1419, Hcc1428, and SUM1315 cell lines were grown in the Kuperwasser lab at Tufts University as previously described [32] and all other cell lines were obtained from ATCC (Manassas, VA, USA). All cell lines were grown as per ATCC’s recommendations and detailed information regarding their origin and culture conditions can be found in Additional file 9.

### Single-genome sequencing

ZR-75-1, MCF-7, T47D, SK-BR-3, Hcc1954, BT20, Hs578T, and MDA-MB-231 breast cancer cells were grown to 90% confluency. RNA was extracted and purified using the RNeasy Mini Kit (Qiagen, Valencia, CA, USA, Cat. No. 74104) and all DNA contamination was removed through DNase treatment (Turbo DNA-*free* Kit, Ambion, Foster City, CA, USA, Cat. No. AM1907). RT reactions were set up as recommended by the manufacturer’s protocol using an oligo(dT) primer (SuperScript III One-Step RT-PCR System, Invitrogen, Carlsbad, CA, USA, Cat. No. 12574-018). The resulting cDNA was serially diluted down to an average of 1/3 genome per sample and amplified using Taq DNA polymerase (Invitrogen, Cat. No. 10342-020). Two forward primers (5′-TTCCTTTACAAAGTTGCGTAAAGC-3′, 5′-GTTGCGTAAAGCCCCCTTAT-3′) and one reverse primer (5′-CACAGACACAGTAACAATCTG-3′), all targeting the HML-2 *env* region, were used in the reaction. The amplified products were gel extracted with the QIAquick Gel Extraction Kit (Qiagen, Cat. No. 28704) and purified samples were sent out for sequencing. The primers used for sequencing were 5′-GACTCCCAGACTATAACCTGTC-3′ and 5′-CGAAGCATCAAAAGCCCA-3′. Sequencing results were BLAT searched in the UCSC Genome Browser [58] to identify expressed proviruses.

### Phylogenetic analysis

The 5′ and 3′ LTR sequence of each provirus of interest was obtained from the UCSC Genome Browser’s RepeatMasker Track [58, 59] and imported as FASTA files into the Molecular Evolutionary Genetics Analysis (MEGA, v6.06) program for alignment using Multiple Sequence Comparison by Log-Expectation (MUSCLE) [60, 61]. Phylogeny of aligned sequences was determined by sequence dissimilarity and a neighbor-joining tree was constructed using a p-distance algorithm. Bootstrap values were determined using 1000 replicate tests.

### Dual-luciferase assay

Primers for LTR amplification were selected using the Primer3 program [62]. Restriction enzyme cleavage sites were appended to the 5′ end of the primer sequences for proper vector ligation. The primers created are listed in Additional file 10. The LTR sequences were PCR-amplified using Taq DNA polymerase. Template DNA was purified from Tera-1 cells using the DNeasy Blood and Tissue Kit (Qiagen, Cat. No. 69504). The amplified sequences were cloned using basic molecular biology techniques and ligated into the multiple cloning region of the pGL4.17[*luc2*/Neo] promoter-less firefly luciferase vector (Promega, Madison, WI, USA, Cat. No. E6721). All constructs were sequenced to check for PCR-induced mutations before transfection. All cell cultures were seeded in triplicate at 100,000 cells/well in a 24-well plate for transfection. Cultures were co-transfected with the pGL4 vector alongside a pRL-SV40 internal control *Renilla* luciferase vector (Promega, Cat. No. E2231) at a 30:1 ratio using Opti-MEM reduced serum media (Gibco, Cat. No. 31985-070) and Lipofectamine 2000 (Thermo Fisher Technologies, Cat. No. 11668-019), as recommended by the manufacturer’s protocol. Post-transfection, cells were incubated at 37 °C for 48 h before lysis and analysis. Luminescence was measured via the dual-luciferase assay system (Promega, Cat. No. E1910) and quantified as relative light units (RLU) on a BioTek Synergy HT plate reader using Gen5 Data Analysis Software (BioTek Instruments, Winooski, VT, USA). Empty vectors as well as non-transfected cells were measured as a control to determine any cell-specific background signal. LTR promoter activity was calculated as *luc2* activity normalized against that of the internal *Renilla* luciferase control signal.

### HML-2 similarity matrices

The sequence of each “full length” (i.e., not solo LTR) HML-2 provirus annotated within the human reference genome (hg19 build) was obtained from the UCSC Genome Browser [58]. These sequences were input into the Clustal Omega program (The European Bioinformatics Institute (EMBL-EBI), Hinxton, Cambridge, UK) [40] to create a multiple sequence alignment using the HHalign algorithm [63] and to create a percent sequence identity matrix. The HML-2 percent expression similarity matrix was created by making pairwise comparisons of significant promoter expression in each of the eighteen cell lines used in our dual-luciferase analysis.

### Transcription factor binding site analysis

The full sequence of each 5′ LTR of interest was imported into MatInspector, a transcription factor binding site prediction software provided by Genomatix [41]. Any site that was identified in more than one provirus was removed from the analysis to produce a list containing all predicted binding sites unique to each LTR. This program also provided information regarding transcription factors that are known to bind to these sites. Transcript abundance levels of these transcription factors in the Hcc1954, HMLE-Ras, HMLE-Her2, and HME cell lines were determined by Cuffdiff analysis of our previous RNA-Seq results. A full description of the study used to obtain these values is detailed in our previous publication [32] and the RNA-Seq data are deposited in the NCBI Gene Expression Omnibus database under Accession Number GSE84275.

Consensus sequences of the HOX-PBX, RFX3, ATF, and RORA binding sites were determined through a separate MEGA alignment. New reporter constructs containing the consensus (non-active) sites were created through IDT’s gBlocks^®^ Gene Fragments synthesis service (Integrated DNA Technologies, Inc., Coralville, IA, USA). These fragments were directly cloned into the pGL4[*luc2*/Neo] firefly luciferase vector and transfected into cell lines as previously described in the Dual-Luciferase Assay section of the Materials and Methods.

The 5′ and 3′ LTRs of each of the nine proviruses of interest were analyzed in an additional MEGA alignment. All unique transcription factor binding sites found in only one LTR were regarded as being “acquired” and any unique binding sites found in both LTRs were characterized as “present at time of insertion”. Sites located in the 7p22.1b and 21q21.1 proviruses were excluded from the analysis since they no longer possess intact 3′ LTRs [4].

The allele frequencies of each unique binding site were calculated from the VCF (Variant Call Format) files of 2504 individuals, as supplied by phase 3 of the 1000 Genomes Project [42]. VCF files were analyzed computationally using VCFtools, by specifying the genomic coordinates (hg19 build) of each site of interest. All sites with an allele frequency of at least 89% were considered to be fixed in the human population. All sites that were classified as polymorphic within the population had allele frequencies of 52% or less. No binding site that we identified had an allele frequency intermediate of those two thresholds, i.e. calculated to be greater than 52% but less than 89%.

The HOX-PBX binding site was further analyzed in several non-human primate reference genomes as supplied by the UCSC Genome Browser [58]. The Denisovan reference genome sequence was obtained from the Denisova High-Coverage Sequence Reads of the Denisova Seq Track. The chimpanzee, gorilla, orangutan, gibbon, and rhesus reference genome sequences were obtained from the Vertebrate Multiz Alignment & Conservation Track.

## Additional files

**Additional file 1: Table S1.** HML-2 transcript levels detected through single-genome sequencing in breast cancer cell lines of varying molecular subtype.

**Additional file 2: Table S2.** HML-2 similarity matrices.

**Additional file 3: Table S3.** HOX-PBX binding site sequences and genomic coordinates (hg19).

**Additional file 4: Table S4.** RFX3 binding site sequences and genomic coordinates (hg19).

**Additional file 5: Table S5.** ATF binding site sequences and genomic coordinates (hg19).

**Additional file 6: Table S6.** RORA binding site sequences and genomic coordinates (hg19).

**Additional file 7: Figure S1.** HML-2 promoter activity is not breast cancer subtype-specific. Total relative 5′ LTR promoter activity levels of fifteen tumorigenic breast cancer cell lines broken down by molecular subtype (luminal, HER2+, and basal-like) as compared to two immortalized HME cell lines. Hormone receptor status and cell lines identified as being each molecular subtype are shown in detail in Table 2. All experiments were conducted in triplicate and data display the mean ± standard deviation.

**Additional file 8: Figure S2.** Allele frequencies of polymorphic HML-2 5′ LTR transcription factor binding sites within each superpopulation. Allele frequencies were determined for the proviruses shown from 2504 individuals from the 1000 Genomes Project and broken down by superpopulation (EAS = East Asian; AMR = Ad Mixed American; AFR = African; EUR = European; SAS = South Asian). The name of the transcription factor binding site as well as the provirus of interest are shown at the top of each graph.

**Additional file 9: Table S7.** Culture methods for cell lines used.

**Additional file 10: Table S8.** Primers used to amplify 5′ LTRs of transfected HML-2 proviruses.
