# Supplementary material for: Promoter expression of HERV-K (HML-2) provirus-derived sequences is related to LTR sequence variation and polymorphic transcription factor binding sites
Source: Retrovirology. 2018 Aug 20;15:57. doi: 10.1186/s12977-018-0441-2 (PMC6102855; doi:10.1186/s12977-018-0441-2)
Supplement: Supplementary file 1 — Additional file 1: Table S1. HML-2 transcript levels detected through single-genome sequencing in breast cancer cell lines of varying molecular subtype. [file 12977_2018_441_MOESM1_ESM.pdf]

Supplementary Table S1. HML-2 transcript levels detected through single-genome sequencing in breast cancer cell lines of varying molecular subtype.

**Provirus**	**Breast Cancer Molecular Subtype**			**Total Number of Transcripts**
	**Luminal**[a]	**HER2/*neu***[b]	**Basal**[c]	
1q22	11	30	28	69
3q12.3	20	17	15	52
21q21.1	2	-	7	9
11p15.4	8	4	1	13
7p22.1	3	7	-	10
3q21.2	2	5	1	8
K105	3	1	1	5
16p11.2	3	-	2	5
22q11.21	-	3	-	3
5p13.3	-	3	-	3

[a]Luminal cell lines: ZR-75-1, MCF-7, T47D; [b]HER2/*neu* cell lines: SK-BR-3, Hcc1954; [c]Basal cell lines: BT20, Hs578T, MDA-MB-231
