# Supplementary material for: Promoter expression of HERV-K (HML-2) provirus-derived sequences is related to LTR sequence variation and polymorphic transcription factor binding sites
Source: Retrovirology. 2018 Aug 20;15:57. doi: 10.1186/s12977-018-0441-2 (PMC6102855; doi:10.1186/s12977-018-0441-2)
Supplement: Supplementary file 2 — Additional file 2: Table S2. HML-2 similarity matrices. [file 12977_2018_441_MOESM2_ESM.pdf]

Supplementary Table S2. HML-2 similarity matrices.

HML-2 Percent Sequence Identity Matrix										
	**1q22**	**3q12.2**	**3q21.2**	**5p13.3**	**7p22.1b**	**8p23.1c**	**11p15.4**	**21q21.1**	**22q11.21**	
**1q22**	100	94	97	97	99	70	69	97	98	**1q22**
**3q12.3**		100	94	95	95	70	69	95	94	**3q12.3**
**3q21.2**			100	97	97	68	68	97	97	**3q21.2**
**5p13.3**				100	97	69	68	97	97	**5p13.3**
**7p22.1b**					100	70	69	97	98	**7p22.1b**
**8p23.1c**						100	95	69	70	**8p23.1c**
**11p15.4**							100	69	69	**11p15.4**
**21q21.1**								100	97	**21q21.1**
**22q11.21**									100	**22q11.21**

HML-2 Percent Expression Similarity Matrix										
	**1q22**	**3q12.2**	**3q21.2**	**5p13.3**	**7p22.1b**	**8p23.1c**	**11p15.4**	**21q21.1**	**22q11.21**	
**1q22**	100	32	95	95	95	68	53	89	89	**1q22**
**3q12.3**		100	37	26	26	63	68	26	37	**3q12.3**
**3q21.2**			100	89	89	74	58	89	89	**3q21.2**
**5p13.3**				100	100	63	47	100	89	**5p13.3**
**7p22.1b**					100	63	47	100	89	**7p22.1b**
**8p23.1c**						100	74	63	74	**8p23.1c**
**11p15.4**							100	47	47	**11p15.4**
**21q21.1**								100	89	**21q21.1**
**22q11.21**									100	**22q11.21**
