# Supplementary material for: Promoter expression of HERV-K (HML-2) provirus-derived sequences is related to LTR sequence variation and polymorphic transcription factor binding sites
Source: Retrovirology. 2018 Aug 20;15:57. doi: 10.1186/s12977-018-0441-2 (PMC6102855; doi:10.1186/s12977-018-0441-2)
Supplement: Supplementary file 3 — Additional file 3: Table S3. HOX-PBX binding site sequences and genomic coordinates (hg19). [file 12977_2018_441_MOESM3_ESM.pdf]

Supplementary Table S3. HOX-PBX binding site sequences and genomic coordinates (hg19).

Proviral LTR	Strand	Genomic Coordinates (hg19)	Sequence
Consensus sequence			AACCCGATT----GTAT
1q22 5'LTR	-	chr1:155605078-155605090	AACCC----GATTGTAT
1q22 3'LTR	-	chr1:155596866-155596878	AACCC----GATTGTAT
3q12.3 5'LTR	+	chr3:101411273-101411289	AACCCGATTGATTGTAC
3q12.3 3'LTR	+	chr3:101419427-101419439	AACCC----GATTGTAC
3q21.2 5'LTR	+	chr3:125609829-125609841	AACCT----GATTGTAT
3q21.2 3'LTR	+	chr3:125618163-125618175	AACCC----GATTGTAT
5p13.3 5'LTR	-	chr5:30495665-30495677	AACCC----GATTGTAT
5p13.3 3'LTR	-	chr5:30487169-30487181	AACCC----GATTGTAT
7p22.1b 5'LTR	-	chr7:4630970-4630982	AACCC----GATTGTAT
8p23.1c 5'LTR	-	chr8:12082912-12082924	AACCC----GATTGTAC
8p23.1c 3'LTR	-	chr8:12074412-12074424	AACCC----GATTGTAC
11p15.4 5'LTR	-	chr11:3477624-3477636	AACCC----AATTGTAC
11p15.4 3'LTR	-	chr11:3469098-3469110	AACCC----GATTGTAC
21q21.1 5'LTR	-	chr21:19941404-19941416	AACCC----AATTGTAT
22q11.21 5'LTR	+	chr22:18926722-18926734	AACCC----GATTGTAT
22q11.21 3'LTR	+	chr22:18934929-18934941	AACCC----GATTGTAT
