# Supplementary material for: Promoter expression of HERV-K (HML-2) provirus-derived sequences is related to LTR sequence variation and polymorphic transcription factor binding sites
Source: Retrovirology. 2018 Aug 20;15:57. doi: 10.1186/s12977-018-0441-2 (PMC6102855; doi:10.1186/s12977-018-0441-2)
Supplement: Supplementary file 4 — Additional file 4: Table S4. RFX3 binding site sequences and genomic coordinates (hg19). [file 12977_2018_441_MOESM4_ESM.pdf]

Supplementary Table S4. RFX3 binding site sequences and genomic coordinates (hg19).

Proviral LTR	Strand	Genomic Coordinates (hg19)	Sequence
Consensus sequence			CTTGTGACCCTGACACATC
1q22 5'LTR	-	chr1:155604875-155604893	CTTGTGACCCTGACACATC
1q22 3'LTR	-	chr1:155596663-155596681	CTTGTGACCCTGACACATC
3q12.3 5'LTR	+	chr3:101411487-101411505	CTTGTGACCATGACACATC
3q12.3 3'LTR	+	chr3:101419635-101419653	CTTGTGACCATGACACATC
3q21.2 5'LTR	+	chr3:125610037-125610055	CTTGTGACCCTGACACATC
3q21.2 3'LTR	+	chr3:125618371-125618389	CTTGTGACCCTGACACATC
5p13.3 5'LTR	-	chr5:30495462-30495480	CTTGTGACCCTGACACATC
5p13.3 3'LTR	-	chr5:30486966-30486984	CTTGTGACCCTGACACATC
7p22.1b 5'LTR	-	chr7:4630767-4630783	CTTGTGACCCTGACACATC
8p23.1c 5'LTR	-	chr8:12082684-12082702	CCTGCTTTCCTAGTATATT
8p23.1c 3'LTR	-	chr8:12082684-12082702	CCTGCTCTCCTATTACATT
11p15.4 5'LTR	-	chr11:3477396-3477414	CCTGCTCTCCTACTACATT
11p15.4 3'LTR	-	chr11:3468868-3468886	CCTGCTCTCCTATTACATT
21q21.1 5'LTR	-	chr21:19941201-19941219	CTTGTGACCCTGACACATC
22q11.21 5'LTR	+	chr22:18926930-18926948	CTTGTGACCCTGACACATC
22q11.21 3'LTR	+	chr22:18935137-18935155	CTTGTGACCCTGACACATC
