# Supplementary material for: Promoter expression of HERV-K (HML-2) provirus-derived sequences is related to LTR sequence variation and polymorphic transcription factor binding sites
Source: Retrovirology. 2018 Aug 20;15:57. doi: 10.1186/s12977-018-0441-2 (PMC6102855; doi:10.1186/s12977-018-0441-2)
Supplement: Supplementary file 5 — Additional file 5: Table S5. ATF binding site sequences and genomic coordinates (hg19). [file 12977_2018_441_MOESM5_ESM.pdf]

Supplementary Table S5. ATF binding site sequences and genomic coordinates (hg19).

Proviral LTR	Strand	Genomic Coordinates (hg19)	Sequence
Consensus sequence			GCCTTAGGGCTGGAG--GTGGGA
1q22 5′LTR	-	chr1:155605040-155605060	GCCTTAGGGCTGGAG--GTGGGA
1q22 3′LTR	-	chr1:155596828-155596848	GCCTTAGGGCTGGAG--GTGGGA
3q12.3 5′LTR	+	chr3:101411318-101411340	GCCTTAGGGCTGGAGGTGTGGGA
3q12.3 3′LTR	+	chr3:101419468-101419488	GCCTTAGGGCTGGAG--GTGGGA
3q21.2 5′LTR	+	chr3:125609870-125609890	GCCTTAGGGCTGGAG--GTGGGA
3q21.2 3′LTR	+	chr3:125618204-125618224	GCCTTAAGGCTGGAA--GTAGGA
5p13.3 5′LTR	-	chr5:30495627-30495647	GCCTTAGGGCTGGAG--GTGGGA
5p13.3 3′LTR	-	chr5:30487131-30487151	GCCTTAGGGCTGGAG--GTGGGA
7p22.1b 5′LTR	-	chr7:4630932-4630952	GCCTTAGGGCTGGAG--GTGGGA
8p23.1c 5′LTR	-	chr8:12082873-12082893	GCCCTGTGATGGAG--GCAAGA
8p23.1c 3′LTR	-	chr8:12074373-12074393	GCCCTGTGGCGGAG--GCGAGA
11p15.4 5′LTR	-	chr11:3477585-3477605	GCCCTGTGACGGAG--GCGAGA
11p15.4 3′LTR	-	chr11:3469059-3469079	GCCCTGTGGCGGAG--GCGAGA
21q21.1 5′LTR	-	chr21:19941366-19941386	GCCTTAGGGCTGGAG--GTGGGA
22q11.21 5′LTR	+	chr22:18926763-18926783	GCCTTAGGGCTGGAG--GTGGGA
22q11.21 3′LTR	+	chr22:18934970-18934990	GCCTTAGGGCTGGAG--GTGGGA
