# Supplementary material for: Promoter expression of HERV-K (HML-2) provirus-derived sequences is related to LTR sequence variation and polymorphic transcription factor binding sites
Source: Retrovirology. 2018 Aug 20;15:57. doi: 10.1186/s12977-018-0441-2 (PMC6102855; doi:10.1186/s12977-018-0441-2)
Supplement: Supplementary file 6 — Additional file 6: Table S6. RORA binding site sequences and genomic coordinates (hg19). [file 12977_2018_441_MOESM6_ESM.pdf]

Supplementary Table S6. RORA binding site sequences and genomic coordinates (hg19).

Proviral LTR	Strand	Genomic Coordinates (hg19)	Sequence
Consensus sequence			GTGTTTGTCTGCTGACCCTCTCCCCACAA
1q22 5’LTR	-	chr1:155604898-155604926	GTGTTTGTCTGCTGACCCTCTCCCCACAA
1q22 3’LTR	-	chr1:155596686-155596714	GTGTTTGTCTGCTGACCCTCTCCCCACAA
3q12.3 5’LTR	+	chr3:101411454-101411482	GTGTTTGTCTGCTGACCCTCTCCCCACTA
3q12.3 3’LTR	+	chr3:101419602-101419630	GTGTTTGTCTGCTGACCCTCTCCCCACTA
3q21.2 5’LTR	+	chr3:125610004-125610032	GTGTTTGTCTGCTGACCCTCTCCCCACAA
3q21.2 3’LTR	+	chr3:125618338-125618366	GTGTTTGTCTGCTGACCCTCTCCCCACAA
5p13.3 5’LTR	-	chr5:30495485-30495513	GTGTTTGTCTGCTGACCCTCTCCCCACAA
5p13.3 3’LTR	-	chr5:30486989-30487017	GTGTTTGTCTGCTGACCCTCTCCCCACAA
7p22.1b 5’LTR	-	chr7:4630790-4630818	ATGTTTGTCTGCTGACCCTCTCCCCACAA
8p23.1c 5’LTR	-	chr8:12082707-12082731	ATGTTT-TTTGTTGAC---CTCCTTATTA
8p23.1c 3’LTR	-	chr8:12074205-12074232	ATGTTT-TTTGTTGACCTTCTCCTTATTA
11p15.4 5’LTR	-	chr11:3477419-3477443	ATGTTT-TTTGTTGAC---CTCCTTAATA
11p15.4 3’LTR	-	chr11:3468891-3468918	ATGTTT-TTTGTTGACCTTCTCCTTATTA
21q21.1 5’LTR	-	chr21:19941224-19941252	GTGTTTGTCTGCTGACCCTCTCCCCACAA
22q11.21 5’LTR	+	chr22:18926897-18926925	GTGTTTGTCTGCTGACCCTCTCCCCACAA
22q11.21 3’LTR	+	chr22:18935104-18935132	GTGTTTGTCTGCTGACCCTCTCCCCACAA
