# Supplementary figures and images for: Promoter expression of HERV-K (HML-2) provirus-derived sequences is related to LTR sequence variation and polymorphic transcription factor binding sites
Source: Retrovirology. 2018 Aug 20;15:57. doi: 10.1186/s12977-018-0441-2 (PMC6102855; doi:10.1186/s12977-018-0441-2)

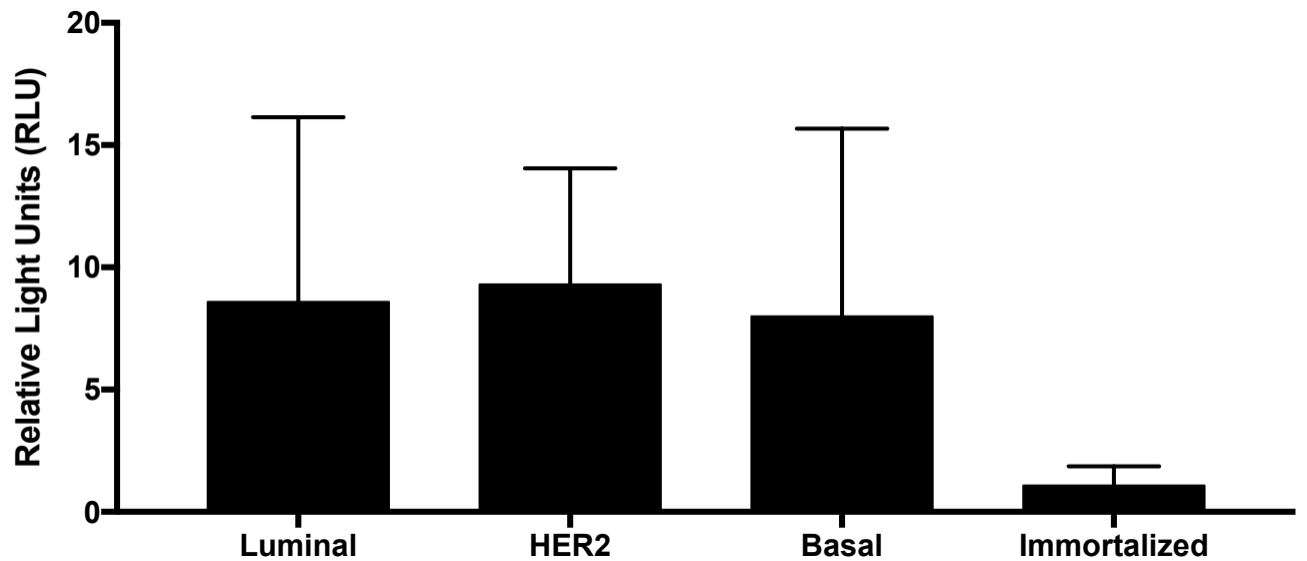

Supplement: Supplementary file 7 — Additional file 7: Figure S1. HML-2 promoter activity is not breast cancer subtype-specific. Total relative 5′ LTR promoter activity levels of fifteen tumorigenic breast cancer cell lines broken down by molecular subtype (luminal, HER2+, and basal-like) as compared to two immortalized HME cell lines. Hormone receptor status and cell lines identified as being each molecular subtype are shown in detail in Table 2. All experiments were conducted in triplicate and data display the mean ± standard deviation. [file 12977_2018_441_MOESM7_ESM.pdf]

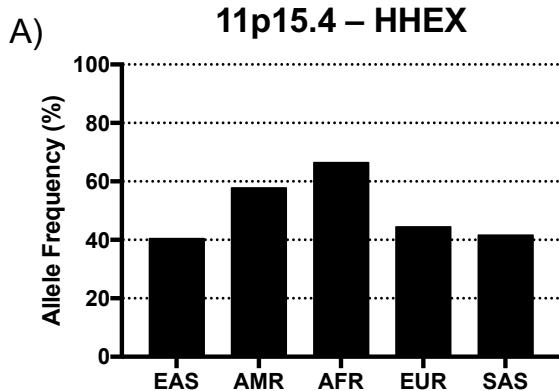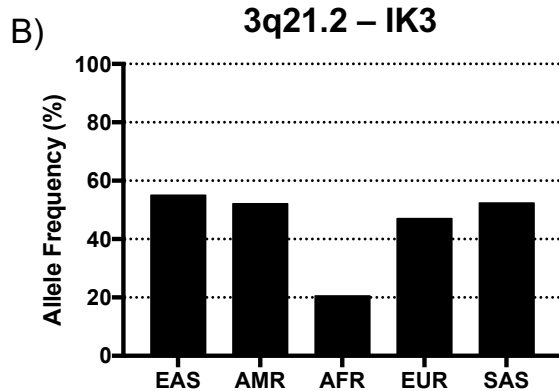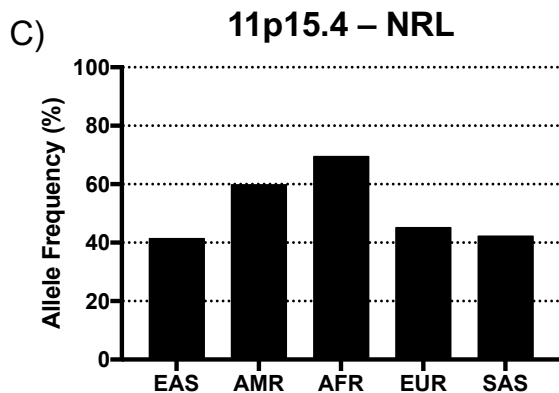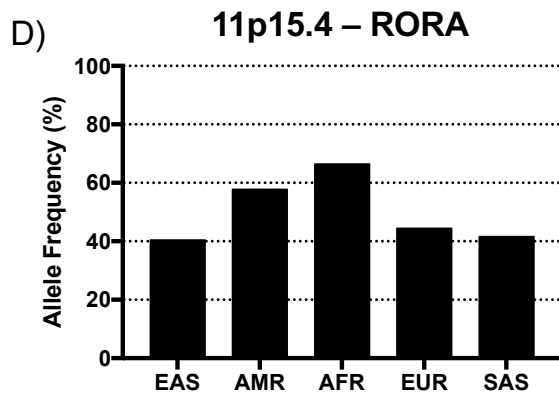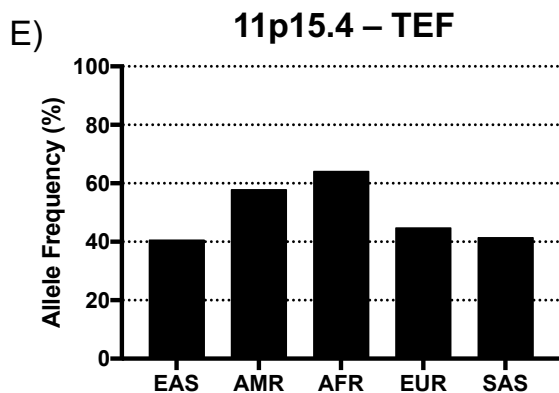

Supplement: Supplementary file 8 — Additional file 8: Figure S2. Allele frequencies of polymorphic HML-2 5′ LTR transcription factor binding sites within each superpopulation. Allele frequencies were determined for the proviruses shown from 2504 individuals from the 1000 Genomes Project and broken down by superpopulation (EAS = East Asian; AMR = Ad Mixed American; AFR = African; EUR = European; SAS = South Asian). The name of the transcription factor binding site as well as the provirus of interest are shown at the top of each graph. [file 12977_2018_441_MOESM8_ESM.pdf]
