# Supplementary material for: Promoter expression of HERV-K (HML-2) provirus-derived sequences is related to LTR sequence variation and polymorphic transcription factor binding sites
Source: Retrovirology. 2018 Aug 20;15:57. doi: 10.1186/s12977-018-0441-2 (PMC6102855; doi:10.1186/s12977-018-0441-2)
Supplement: Supplementary file 9 — Additional file 9: Table S7. Culture methods for cell lines used. [file 12977_2018_441_MOESM9_ESM.pdf]

Supplementary Table S7. Culture methods for cell lines used.

Base Medium	Supplements	Cell Lines
MEGM (Mammary Epithelial Cell Growth Medium)[a]	4 mg/ml bovine pituitary extract[a]<br>1 mg/ml human epidermal growth factor[a]<br>1 mg/ml insulin[a]<br>1 mg/ml hydrocortisone[a]<br>1 mg/ml gentamicin sulfate/amphotericin-B[a]<br>0.1 mg/ml cholera toxin[b]	HME[l]<br>HMLE-Her2[m]<br>HMLE-Ras[n]<br>MCF-10A
DMEM[c]	10% FBS[d]<br>1% Pen-Strep[e]	MDA-MB-361<br>MDA-MB-231<br>MCF-7<br>BT20<br>BT474<br>Hs578T
Ham's F-12[f]	5% FBS[d]<br>1 µg/ml hydrocortisone[g]<br>5 µg/ml insulin[h]	SUM149<br>SUM159
Ham's F-12[f]	5% FBS[d]<br>10 ng/ml human epidermal growth factor[i]<br>5 µg/ml insulin[h]	SUM1315
RPMI 1640[j]	10% FBS[d]<br>1% Pen-Strep[e]	Hcc1428<br>Hcc1419<br>Hcc1954<br>ZR-75-1<br>T47D
McCoy's 5A[k]	10% FBS[d]<br>1% Pen-Strep[e]	SK-BR-3
McCoy's 5A[k]	15% FBS[d]<br>1% Pen-Strep[e]	Tera-1

[a]MEGM BulletKit, Lonza, Walkersville, MD, USA, Cat. No. CC-3150; [b]Sigma, St. Louis, MO, USA, Cat. No. C8052; [c]Gibco, Carlsbad, CA, USA, Cat. No. 10566-036; [d]Atlanta Biologicals, Norcross, GA, USA, Cat. No. S11195; [e]Gibco, Cat. No. 15140122; [f]Gibco, Cat. No. 31765-035; [g]Sigma, Cat. No. H4001; [h]Sigma, Cat. No. I2643; [i]Sigma, Cat. No. E9644; [j]Gibco, Cat. No. 61870-036; [k]Gibco, Cat. No. 16600-082; [l]hTERT-immortalized, non-transformed; [m]hTERT-immortalized, SV40 transformed, *ERBB2* overexpression; [n]hTERT-immortalized, SV40 transformed, *HRAS* overexpression. A detailed description of the production of the HME, HMLE-Her2, and HMLE-Ras cell lines can be found in Montesion *et al* [32].
