# Supplementary material for: Promoter expression of HERV-K (HML-2) provirus-derived sequences is related to LTR sequence variation and polymorphic transcription factor binding sites
Source: Retrovirology. 2018 Aug 20;15:57. doi: 10.1186/s12977-018-0441-2 (PMC6102855; doi:10.1186/s12977-018-0441-2)
Supplement: Supplementary file 10 — Additional file 10: Table S8. Primers used to amplify 5′ LTRs of transfected HML-2 proviruses. [file 12977_2018_441_MOESM10_ESM.pdf]

Supplementary Table S8. Primers used to amplify 5' LTRs of transfected HML-2 proviruses.

Provirus[a]	Amplifed Region (hg19)	BLAST Results (hg19)	Amplifed Region (hg38)	BLAST Results (hg38)	Primer Sequences
1q22	chr1:155,604,094-155,605,800	100.0%	chr1:155,634,303-155,636,009	100.0%	F: 5'-ATTATAGAGCTCCGTTGACTGAGCCATTACCG-3'<br>R: 5'-ATTATAGGTACCTAAAATCCAGCAGCCCAGGA-3'
3q12.3	chr3:101,410,385-101,412,176	100.0%	chr3:101,691,541-101,693,332	100.0%	F: 5'-ATTATAGGTACCAAGGAGGCTGAGCAGATGAG-3'<br>R: 5'-ATTATTAAGCTTTTCCAGGGGCATCAGAAACT-3'
3q21.2	chr3:125,609,085-125,610,870	100.0%	chr3:125,890,242-125,892,027	100.0%	F: 5'-ATTATAGGTACCTCACCACCAACCCAGCTAAT-3'<br>R: 5'-ATTATAGAGCTCTCTGGCGGTTGGGTCTTATT-3'
5p13.3	chr5:30,494,698-30,496,674	100.0%	chr5:30,494,591-30,496,567	100.0%	F: 5'-ATTATAGAGCTCCGGCTCTGCTACATATTCGC-3'<br>R: 5'-ATTATAGGTACCGGACACATACACCCTCCCAA-3'
7p22.1b	chr7:4,638,489-4,640,273	100.0%	chr7:4,598,858-4,600,642	100.0%	F: 5'-ATTATAGAGCTCCGTTGACTGAGCCATTACCG-3'<br>R: 5'-ATTATAGGTACCAATACCCACAGCACCCAAGA-3'
8p23.1c	chr8:12,082,194-12,083,779	100.0%	chr8:12,224,685-12,226,270	100.0%	F: 5'-ATTATAGAGCTCAACCATGGGCGGAATTGTTC-3'<br>R: 5'-ATTATAGGTACCAAGAAGTCCACCTGCCTCAA-3'
11p15.4	chr11:3,476,909-3,478,491	100.0%	chr11:3,455,679-3,457,261	100.0%	F: 5'-ATTATAGAGCTCAACCATGGGCGGAATTGTTC-3'<br>R: 5'-ATTATAGGTACCAAGAAGTCCACCTGCCTCAA-3'
21q21.1	chr21:19,940,483-19,942,138	100.0%	chr21:18,568,165-18,569,820	100.0%	F: 5'-ATTATAGAGCTCGGTTTAGACTCTGGTGGCCT-3'<br>R: 5'-ATTATAGGTACCTCATGCAGCCTGTAAGTGGA-3'
22q11.21	chr22:18,926,085-18,927,711	100.0%	chr22:18,938,572-18,940,198	100.0%	F: 5'-ATTATAGGTACCTGCCTCAACCTCCCAAGTAG-3'<br>R: 5'-ATTATAGAGCTCCGGCTCTGCTACATATTCGC-3'

[a]All provirus constructs were validated by PCR to ensure 100% identity with the human reference genome at the genomic coordinates provided in the amplified region (hg19) column and amplified region (hg38) column.
